# Cost of illness of breast cancer in low- and middle-income countries: a systematic review

**DOI:** 10.1186/s13561-024-00536-0

**Published:** 2024-07-22

**Authors:** Siew Wei Yeong, Sit Wai Lee, Siew Chin Ong

**Affiliations:** 1 School of Pharmaceutical Sciences, Universiti Sains Malaysia, Georgetown, Penang Malaysia; 2 Malaysian Health Technology Assessment Section, Medical Development Division, Ministry of Health Malaysia, Putrajaya, Malaysia

**Keywords:** Cost of illness, Breast cancer, Direct medical costs, Direct non-medical costs, Productivity loss, Health economics

## Abstract

This systematic review explores the cost of illness (COI) studies on breast cancer in low- to middle-income countries (LMICs). Studies in Cochrane, Proquest Thesis, PubMed and Scopus were considered. The reporting criteria were evaluated using the Consolidated Health Economic Evaluation Reporting Standards (CHEERS) 2022 statement. Studies must (1) be peer-reviewed, (2) report cost data, and (3) be full-text articles. Non-English articles were excluded. Twelve studies were included. The identified costs were made constant to 2022 USD values for reporting and comparison across studies. Annual costs per patient varied from $195 to $11,866 direct medical costs, $201 to $2233 direct non-medical costs and $332 to $26,390 productivity losses were reported. Cost differences were due to the cost types and components in each study. Only three COI studies reported sensitivity analysis and discount rates. Hence, it is recommended that future COI studies include an analysis of correlation between cost components and other variables.

**Supplementary Information:**

The online version contains supplementary material available at 10.1186/s13561-024-00536-0.

## Highlights

Direct medical costs with hospitalisation were reported as the highest cost of breast cancer among the LMICs.

Direct non-medical costs and productivity losses added to the financial burden of breast cancer patients.

COI studies without sensitivity analysis cannot predict breast cancer outcomes.

Correlation analysis between cost components and other variables in future COI studies could provide more information for decisions on healthcare resource allocation and planning.

**Supplementary Information:**

The online version contains supplementary material available at 10.1186/s13561-024-00536-0.

## Background

Female breast cancer is the most frequently diagnosed cancer (11.7% of all cancers worldwide), besides being the primary reason that women succumb to cancer [1]. Breast cancer incidence and mortality age-adjusted rates per 100,000 populations are rapidly increasing worldwide, with rate differences between LMICs and high-income countries (HICs).

HICs have a significantly higher incidence of breast cancer (55.9 age-standardised rate (ASR) per 100,000 female population) compared to LMICs (29.7 ASR per 100,000 female population). However, the mortality rate of breast cancer in HICs is lower than in LMICs (12.8 vs. 15 ASR per 100,000 female population). In addition, breast cancer is diagnosed at later stages in LMICs than in HICs due to less awareness and less accessibility to health care [2, 3]. Growing rates of cancer in LMICs further strain the economic and healthcare systems in these countries and present special difficulties in extrapolating the cancer control experience in HICs to LMICs. Thus, COI studies would provide useful insights to decide on the allocation of healthcare resources for the early detection of breast cancer.

The high incidence and cost of female breast cancer impose a considerable burden on the healthcare systems of the LMICs [4–6]. COI studies seek to identify and quantify all expenses associated with a health condition and reflect the financial burden of a given population [7, 8] as they provide valuable information to authorities in the process of forming public health policies. The purpose of COI studies is to itemise the costs of healthcare resources, followed by valuing the cost items and summing up the costs.

Healthcare costs are divided into direct medical costs, direct non-medical costs, indirect costs and intangible costs [9]. Opportunity costs arise when funds are allocated for a particular healthcare resource and cannot be used for other resources. Health economics makes an effort to analyse healthcare costs while taking opportunity costs into account when allocating resources. To determine the usage of healthcare resources, direct medical costs are typically obtained. Patients’ medical bills or medical records are common sources of data collection for direct medical costs. Unit costs of healthcare resources used can be obtained from the published costs in gazette public documents or from the market price of health resources in a country. Direct non-medical costs are expenses incurred by patients during the course of their illness that are not medicine in nature. Examples include the cost of lodging, transportation, nursery or in-home care while receiving medical treatment. These are usually obtained from patient surveys and can often depend on individual household structure and distance to the treatment sites. The other cost category is the indirect cost, which is usually measured as productivity loss due to illness or death. The less reported costs in published literature are the intangible costs, such as pain, suffering, anxiety and depression caused by the illness. It is often difficult to quantify intangible costs. Stakeholders receive more important information from studies that report all cost types and offer in-depth analysis.

Original research and data from LMICs are particularly valuable in providing accurate insights into the disease burden in these countries. This systematic review intends to identify breast cancer COI studies from LMICs. The quality of COI study is important to help stakeholders determine the comprehensiveness, accuracy and validity of the cost types and cost data. In a consensus guideline for the critical appraisal of COI studies [10], researchers have been urged to justify the inclusion or exclusion of certain cost types and data. A systematic review should include findings on several quality matters, such as the research design, research questions and objectives, perspective of the study, cost allocation, cost methodology, uncertainty and sensitivity analysis, as well as subgroup analysis, for an accurate report on the disease burden of an illness across the intended comparison countries [11, 12].

This study aims to systematically review published COI studies of LMICs for cost itemisations, cost valuation and cost analysis. It analyses the cost methods used and determines methodological areas which could be enhanced for comparable studies across countries. This study is also aimed at analysing the reporting quality of the included studies.

## Methods

This systematic review was planned and reported using the Cochrane Handbook as a general reference. The Preferred Reporting Items for Systematic Review and Meta-Analysis Protocol (PRISMA) checklist were engaged for reporting this systematic review, including the PRISMA 2020 Flow Diagram.

### Study design

The literature on the cost of illness studies of breast cancer in LMICs was thoroughly reviewed and served as a general framework for this systematic review.

### Search strategy

A search of the literature was conducted using Cochrane, PubMed, Proquest Thesis and Scopus. Further search was conducted from the reference list of the included studies. The search was limited to studies published up to 25 Dec 2023. The title and abstract of the peer-reviewed journal articles were first screened using synonyms and MeSH terms. Main areas for the search included: (a) Breast cancer, (b) Cost of illness and (c) LMICs. A systematic literature search was conducted with keywords “breast neoplasm” and combined with “cost of illness” from MESH terms.

### Eligibility criteria

#### Inclusion criteria

COI studies with original data, costs and full texts were included in this review.

#### Exclusion criteria

COI studies from non-LMICs and studies in languages other than English were excluded due to limited resources and time. Unfamiliarity with the language may create bias in the review process among the reviewers. Studies on economic evaluation that were not COI studies were excluded to focus on comprehensive COI reports with details.

### Study selection

For the review process, the titles and abstracts were read by two reviewers to screen if the study meets the inclusion criteria. Should the two reviewers have differing opinions, they would further examine the review and consent to resolve it. Nevertheless, if the two reviewers were unable to come to an agreement, a third reviewer would be consulted to contribute to the discussion before a decision was made. Where the titles and abstracts were unclear, a flexible approach was used to include these studies in the more detailed review. Full text was retrieved for further analysis from the screened and included studies.

### Data extraction

#### Cost methodologies

The study design and study characteristics were listed as reported in the selected studies. Studies with a prevalence approach estimate COI at a specific time point, regardless of when the illness was first diagnosed. On the other hand, the incidence approach included the cost of illness from diagnosis and either over a person’s lifetime or over a predetermined period. The included studies in this review were analysed to see if they are adopting either a prevalence, incidence or mixed approach of cost methodologies.

#### Cost perspectives

The study perceptive of the selected studies was reported accordingly.

#### Cost of breast cancer and cost disaggregation

The total cost of breast cancer with its types of costs and cost components were reported when indicated in the selected studies. Campbell and Cochrane Economics Methods Group Evidence for Policy and Practice Information and Coordination Centre (CCEMG - EPPI-Centre) cost converter was used to convert the identified costs in the selected studies to constant 2022 United States Dollars (USD) values for reporting and comparison [13]. Meanwhile, time horizon, discount rates and sensitivity analysis were reported if indicated in the included studies.

### Quality assessment for reporting

Several checklists are available to assess the methodological quality or the reporting quality of COI studies [14], which differ depending on whether the checklist is utilised for methodological quality or more towards reporting quality. Chiou et al. [15] discussed the importance of a simple and expert-based grading system which appraises economic evaluation studies with a focus on the criteria for full economic evaluation (including cost-effectiveness analysis). For this systematic review, consensus-based guidelines specific to COI studies by Schnitzler et al. [10] were used for the critical appraisal of the included studies. In addition, the Consolidated Health Economic Evaluation Reporting Standard (CHEERS) 2022 was used to assess the reporting criteria of the selected studies. [16] Each study was evaluated against the CHEERS 2022 criteria for looking into the reporting quality of the COI studies. All 28 items were assessed except items 11, 12, 13, 16, 17 and 19. Items 11, 12 and 13 are not applicable to COI studies as health outcomes with comparators that were not in the methodology of COI studies. In addition, items 16 and 17 are only applicable to health economics model-based studies. Item 19 was not included as it looks at the distribution effects by social variables applicable to intervention-based studies, not for COI studies in this review.

## Results

### Literature search

**Fig. 1:**
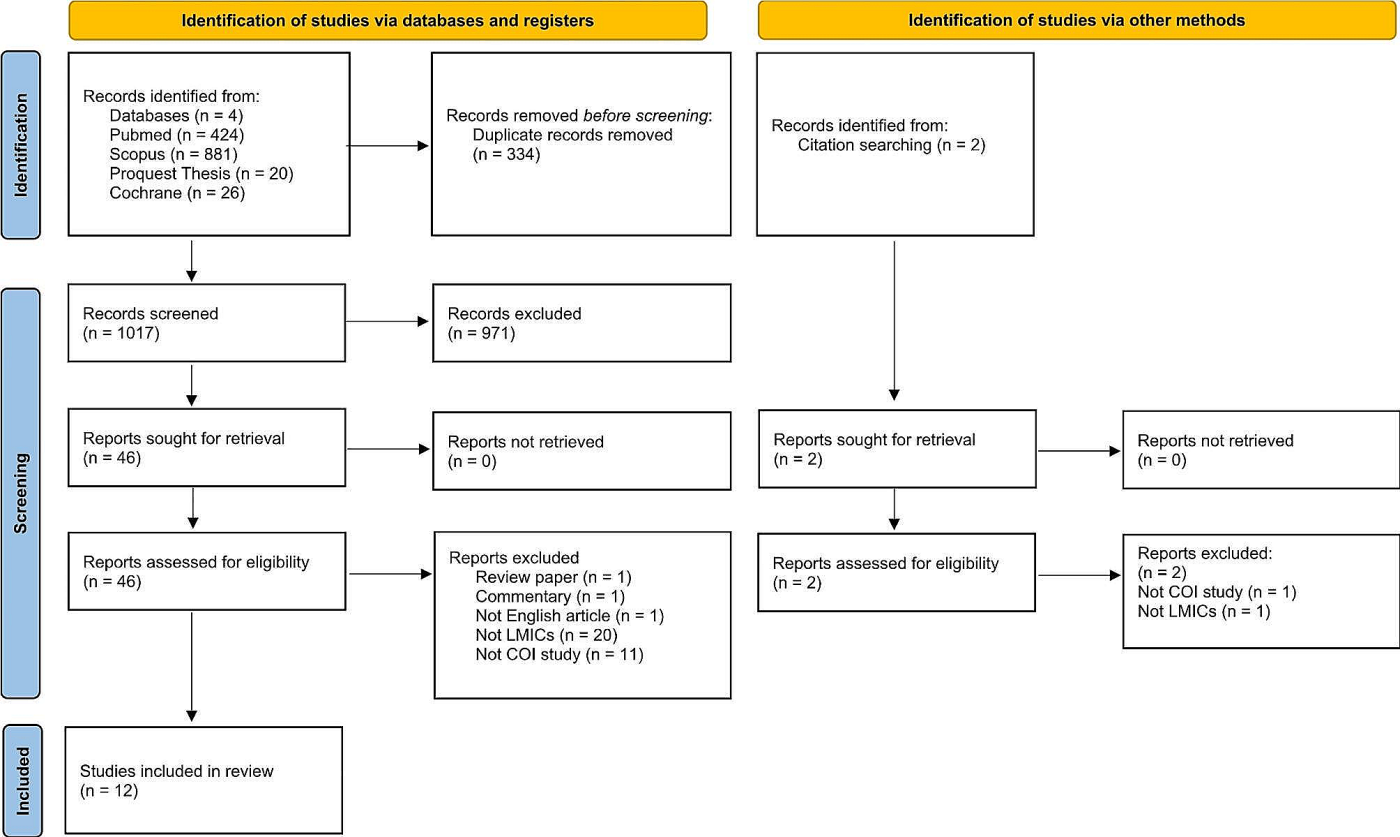
The Preferred Reporting Items for Systematic Reviews and Meta-Analysis (PRISMA) 2020 flow diagram LMICs, low to middle income countries; COI, cost of illness

The early literature search resulted in 1351 studies published by 25 Dec 2023 (Fig. 1). Duplicates were removed, resulting in 1017 studies for screening of titles and abstracts. Twelve studies from LMICs were included after 46 full-text publications were retrieved and reviewed. Further information can be obtained from the Supplementary Material 1: Search Report.

### Study design and study characteristics

The final selection included COI studies of breast cancer in the following countries: Brazil (*n* = 1) [17], China (*n* = 2) [18, 19], Haiti (*n* = 1) [20], India (*n* = 2) [21, 22], Iran (*n* = 3) [23–25], Pakistan (*n* = 1) [26], Vietnam (*n* = 1) [27] and Papua New Guinea [28]. Most studies were from lower-middle-income countries (75%), while the rest were from upper-middle-income countries (25%) (Table 1). None were from the low-income countries (LICs). Over half the studies (*n* = 8) consisted of a medium-sized study population of between 100 and 200, while two studies with large populations comprised databases from health registries or national insurance. Six studies were conducted as incidence-based studies [19–22, 26, 27], whereas the other six studies were prevalence-based [17, 18, 23–25, 28].

**Table 1:** Study characteristics

Study design	Number of studies (%)	Studies
Prospective	3 (25%)	Mahmood et al., 2018; O’Neill et al., 2015; Pakseresht et al., 2011
Retrospective	9 (75%)	Afkar et al., 2020; Afkar et al., 2021; Jalali et al., 2023; Lan et al., 2013; Rezende et al., 2021; Shankar et al., 2018; Umo et al., 2023; Zhao et al., 2022; Zheng et al., 2022
**Setting**		
Hospital patient records	10 (84%)	Afkar et al., 2020; Afkar et al., 2021; Jalali et al., 2023; Mahmood et al., 2018; O’Neill et al., 2015; Pakseresht et al., 2011; Shankar et al., 2018; Lan et al., 2013; Umo et al., 2023; Zhao et al., 2022
National Health Registry	1 (8%)	Zheng et al., 2022
National Health Survey	1 (8%)	Rezende et al., 2021
**Country income**		
Lower middle income	9 (75%)	Afkar et al., 2020; Afkar et al., 2021; Jalali et al., 2023; Lan et al., 2013; Mahmood et al., 2018; O’Neill et al., 2015; Pakseresht et al., 2011; Shankar et al., 2018; Umo et al., 2023
Upper middle income	3 (25%)	Rezende et al., 2021; Zhao et al., 2022; Zheng et al., 2022
**Study population size**		
< 100	2 (17%)	O’Neill et al., 2015; Umo et al. 2023
100–200	8 (66%)	Afkar et al., 2020; Afkar et al., 2021; Jalali et al., 2023; Lan et al., 2013; Mahmood et al., 2018; Pakseresht et al., 2011; Shankar et al., 2018; Zhao et al., 2022
Large population	2 (17%)	Rezende et al., 2021; Zheng et al., 2022
**Study perspective**		
Societal	4 (33%)	Afkar et al., 2021; Jalali et al., 2023; O’Neill et al., 2015; Pakseresht et al., 2011
Payer	8 (67%)	Afkar et al., 2020; Lan et al., 2013; Mahmood et al., 2018; Rezende et al., 2021; Shankar et al., 2018; Zhao et al., 2022; Zheng et al., 2022; Umo et al., 2023
**Costing methodology**		
Bottom-up	7 (58%)	Afkar et al., 2020; Afkar et al., 2021; Jalali et al., 2023; Lan et al., 2013; O’Neill et al., 2015; Pakseresht et al., 2011; Umo et al., 2023
Top-down	3 (25%)	Rezende et al., 2021; Zhao et al., 2022; Zheng et al., 2022
Mixed	2 (17%)	Mahmood et al., 2018; Shankar et al., 2018
**Epidemiological component**		
Incidence	7 (58%)	Lan et al., 2013; Mahmood et al., 2018; O’Neill et al., 2015; Pakseresht et al., 2011; Shankar et al., 2018; Umo et al., 2023; Zhao et al., 2022
Prevalence	5 (42%)	Afkar et al., 2020; Afkar et al., 2021; Jalali et al., 2023; Rezende et al., 2021; Zheng et al., 2022

### Cost perspective of the included studies

The healthcare payer perspective (*n* = 8, 67%) was the most frequent methodological characteristic of the included studies. Details are provided in Table 2. Three studies followed a prospective approach, whereas the remaining studies adopted a retrospective approach. The three prospective studies collected cost data through patient surveys. Two studies from India [21, 22] reported the costs in categories of ranges, which renders the results less meaningful.

### Cost of breast cancer and cost disaggregation

Table 3 presents the annual cost per patient, ranging from $809 (SD: 605) (Lan et al. [27], Vietnam) to $27,122 (SD: 14,948) (Afkar et al. [24], Iran). The inclusion of hospitalisation costs increased the direct costs to the upper range. In addition, studies in the upper middle-income countries with a top-down approach in cost data methodology reported the highest COI. [17, 18] None of the studies compared the COI to the country’s gross domestic product (GDP). Most of the studies included cost disaggregation in the cost data reporting, except Shankar et al. [22] and Zheng et al. [18], which did not specify the cost components. Shankar et al. did not specify the means or median in the cost data but reported as a range.

#### Direct medical costs

Direct medical costs ranged from $195 (range: 171–297) (O’Neill et al. [20], Haiti) to $11,866 (SD: 23,000) (Afkar et al. [24], Iran) (Table 3). Afkar et al. [24] reported the highest direct costs reported based on a private hospital in Iran. In the same study, the direct cost was reported as $4676 in public hospitals, a difference of 61% between the two settings. Hospitalisation consisted of the most costs in both settings. According to studies conducted by Pakseresht et al. (India) [21] and Lan et al. (Vietnam), medications accounted for 44–78% of the direct medical costs.

#### Direct non-medical costs

Five studies reported transportation, food and lodging costs as direct non-medical costs (Table 3). [20, 21, 24–26] Direct non-medical costs ranged from $201 (SD: 827) (Afkar et al. [24], Iran) to $2233 (SD: 2108) (Jalali et al. [25], Iran). In contrast, accommodation and food contributed to higher direct non-medical costs than transportation, which represented a smaller portion.

#### Indirect costs

Mahmood et al. [26], with data from India, reported indirect costs with a productivity loss of $332 (SD: 631) during the duration of the treatment cycle, while Afkar et al. [23]. in Iran reported an annual income loss of $26,390 (SD: 21,997) among the breast cancer patients in a public hospital setting (Table 2). Productivity loss was higher than the total direct medical costs in a public hospital setting, as reported by Afkar et al. (Table 3) [23]. In addition, only two studies included sensitivity analysis in their methodology [24, 27]. Six studies reported costs for treatment cycles; however, only two adopted a discount rate of 3% [24, 27].

**Table 2:** Cost data and methodology

Author	Year	Country	Cost data duration	Sensiti-vity analysis	Discount rate	Perspective	Cost method	Cost unit	Direct medical costs*	Direct non-medical costs*	Indirect costs*
Pakseresht et al. [21]	2011	India	6 months	None	None	Societal	Bottom-up	Cost per patient	515 (0–3436)	619 (0–3093)	NA
Lan et al. [27]	2013	Vietnam	5 years	Yes	3%	Payer	Bottom-up	Annual cost per patient	809 (SD 605)	NA	NA
O’Neill et al. [20]	2015	Haiti	Treatment cycle	None	None	Societal	Bottom-up	Out-of-pocket cost per patient per treatment cycle	195 (171–297)	676 (502–974)	NA
Shankar et al. [22]	2018	India	Treatment cycle	None	None	Payer	Mixed	Cost per patient per treatment cycle	825 (172–858)	NA	NA
Mahmood et al. [26]	2018	Pakistan	Treatment cycle	None	None	Payer	Mixed	Cost per patient per treatment cycle	1534 (SD 1536)	378 (SD 365)	332 (SD 631)
Afkar et al. [24]	2020	Iran	Treatment cycle	None	3%	Payer	Bottom-up	Cost per patient per treatment cycle	4808**	NA	NA
Afkar et al. [23]	2021	Iran	One year (private hospital)	Yes	None	Societal	Bottom-up	Annual cost per patient in private hospital	11,866 (SD 23,000)	201 (SD 827)	2208 (SD 5821)
			One year (public hospital)	as above	as above	as above	as above	Annual cost per patient in public hospital	4676 (SD 8005)	401 (SD 1063)	26,390 (SD 21,997)
Rezende et al. [17]	2021	Brazil	Annual cost	None	None	Payer	Top-down	Cost for country (2015–2017) (Brazil)	377 million**	NA	NA
Zheng et al. [18]	2022	China	Treatment cycle	None	None	Payer	Top-down	Annual BC cost for city (Dalian, China)	78 million**	NA	NA
Zhao et al. [19]	2022	China	Treatment	None	None	Payer	Top-down	Cost per patient per treatment	952 (708–1303)	NA	NA
Jalali et al. [25]	2023	Iran	Treatment cycle	None	None	Payer	Bottom-up	Cost per patient	9060 (SD 6761)	2233 (SD 2108)	1525 (SD 3152)
Umo et al. [28]	2023	Papua New Guinea	Treatment cycle	None	None	Payer	Bottom-up	Cost per patient	7248**	NA	NA

*Total cost was reported as medians with ranges or means with standard deviation (SD) per patient unless specified

Costs were stated in USD for 2022 based on CCEMG – EPPI-Centre Cost Converter; Costs rounded to the nearest whole numbers

NA, not applicable; SD, standard deviation

** Cost range or SD was not reported in original study

**Table 3:** Cost components of selected studies

Author	Total cost*	Direct cost	Inpatient	Follow-up visits	Surgery	Radiation	Labs	Medica- tions	Direct non-medical cost	Home-help	Childcare	Transpor-tation	Accommodation	Indirect cost
Pakseresht et al. [21]	1146 (0–6186)	**515** **(0–3436)**	NR	NR	NR	NR	29 (0–745)	229 (0–3436)	**619** **(0–3093)**	NR	NR	344 (0–2749)	115 (0–1947)	NA
Lan et al. [27]	809 (SD 605)	**809** **(SD 605)**	34 (SD 16)	471 (SD 344)	108 (SD 42)	30 (SD 50)	NR	630 (SD 996)	NA	NA	NA	NA	NA	NA
O’Neill et al. [20]	880 (298–8865)	**195** **(171–297)**	NR	NR	NR	NR	NR	NR	**676** **(502–974)**	NR	NR	NR	NR	NA
Shankar et al. [22]	825 (172–858)	**825** **(172–858)**	NR	NR	NR	NR	NR	NR	NA	NA	NA	NA	NA	NA
Mahmood et al. [26]	2243 (SD 2106)	**1534** **(SD 1936)**	NR	NR	281 (SD 413)	17 (SD 502)	299 (SD 196)	450 (SD 662)	**378** **(SD 365)**	24 (SD 98)	2.86 (SD 31)	361 (SD 356)	16 (SD 67)	**332** **(SD 631)**
Afkar et al. [24]	4808**	4808**	2081	553	749	NIL	403	1022	NA	NA	NA	NA	NA	NA
Afkar et al. [23]	private hospital 14,122 (SD 24,241)	**11,866** **(SD 23,000)**	2161 (SD 1110)	673 (SD 1322)	1157 (SD 720)	649 (SD 1582)	2869 (SD 8785)	897 (SD 1417)	**201** **(SD 827)**	NR	NR	177 (SD 815)	12 (SD 148)	**2208** **(SD 5821)**
	public hospital 27,122 (SD 14,948)	**4676** **(SD 8005)**	1476 (SD 1830)	118 (SD 378)	230 (SD 396)	105 (SD 160)	502 (SD 1110)	313 (SD 626)	**401** **(SD 1063)**	NR	NR	295 (SD 827)	1086 (SD 673)	**26,390** **(SD 21,997)**
Rezende et al. [17]	377 million (national prevalence)	**377 million**	69 million	NR	NR	53 million	NR	256 million	NA	NA	NA	NA	NA	NA
Zheng et al. [18]	78 million (Dalian city)	**N**A	NA	NA	NA	NA	NA	NA	NA	NA	NA	NA	NA	**NA**
Zhao et al. [19]	952 (708–1303)	**952** **(708–1303)**	NR	NR	NR	NR	NR	NR	NA	NA	NA	NA	NA	**NA**
Jalali et al. [25]	12,818 (SD 8323)	**9060** **(SD 6761)**	2264 (SD 1079)	243 (SD 5495)	NR	3594 (SD 3846)	598 (SD 320)	859 (SD 917)	**2233** **(SD 2108)**	125 (SD 461)		873 (SD 694)	1525 (SD 3152)	**1525** **(SD 3152)**
Umo et al. [28]	7248**	7248**	NR	NR	NR	NR	NR	NR	NA	NA	NA	NA	NA	NA

*Total cost was reported as mean with ranges or mean with standard deviation (SD) per patient unless specified

Costs were stated in USD for 2022 based on CCEMG – EPPI-Centre Cost Converter; Costs rounded to the nearest whole numbers

NA, not applicable; NR, not recorded; SD, standard deviation

** Cost range or SD was not reported in original study

**Table 4:** Consensus-based checklist of critical appraisal of COI study*

Items						Rezende et al. [17]		Zheng et al. [18]		Zhao et al. [19]		O’Neill et al. [20]		Pakseresht et al. [21]		Shankar et al. [22]		Afkar et al. [23]		Afkar et al. [24]		Jalali et al. [25]		Mahmood et al. [26]	Lan et al. [27]	Umo et al., [28]
**Study characteristics**																										
**Research question or objective**: Is a well-defined question or objective stated?						Y		Y		Y		Y		Y		Y		Y		Y		Y		Y	Y	Y
**Population**: Is the study population described?						Y		Y		Y		Y		Y		Y		Y		Y		Y		Y	Y	Y
**Perspective**: (a) Is the chosen perspective stated?						Y		Y		Y		Y		Y		Y		Y		Y		Y		Y	Y	Y
**Perspective**: (b) If so, is the chosen perspective justified?						Y		Y		Y		Y		Y		Y		Y		Y		Y		Y	Y	Y
**Methodology and cost analysis**																										
**Epidemiological approach**: Is the epidemiological approach reported?						P		UC		UC		N		P		UC		Y		Y		Y		NC	Y	Y
**Costing approach**: Is the costing approach reported?						Y		UC		UC		UC		N		N		Y		Y		Y		NC	Y	Y
**Data collection**: Is the data collection approach reported?						Y		Y		Y		Y		Y		Y		Y		Y		Y		Y	Y	Y
**Identification of resources**: (a) Are all components of resource use identified that are relevant to the condition/disease, population, intervention, study objectives and study perspective?						P		UC		P		P		P		UC		P		P		P		P	P	P
**Identification of resources**: (b) If not, is a justification provided for excluding relevant components of resource use?						N		N		N		N		N		N		N		N		N		N	N	N
**Measurement of resources**: (a) Are all identified and included components of resource use measured?						Y		Y		Y		Y		UC		UC		Y		Y		Y		Y	Y	Y
**Measurement of resources**: (b) If not, is a justification provided for not measuring certain components of resource use?						NA		NA		NA		NA		N		N		NA		NA		NA		NA	NA	NA
**Valuation of resources**: (a) Are all included components of usage valued in monetary terms?						Y		Y		Y		Y		Y		N		N		N		N		N	P	N
**Valuation of resources**: (b) If not, is a justification provided for not valuing certain components of resource use?						NA		NA		NA		NA		NA		N		N		N		N		N	N	N
**Time horizon**: (a) Is the chosen time horizon specified?						N		P		N		P		Y		N		Y		Y		N		N	Y	N
**Time horizon**: (b) If so, is the chosen time horizon justified?						NA		P		NA		P		P		NA		N		N		NA		NA	NC	NA
**Discounting**: (a) Are future costs discounted?						N		N		N		N		N		N		Y		Y		N		N	Y	N
**Discounting**: (b) If so, is justification provided for the discount rate?						NA		NA		NA		NA		NA		NA		Y		Y		NA		NA	Y	NA
**Sensitivity**: (a) Are all variables whose values are uncertain subjected to sensitivity analysis?						N		N		N		N		N		N		N		Y		N		N	Y	N
**Sensitivity**: (b) If so, is a justification provided for which variables are subjected to sensitivity analysis?						NA		NA		NA		NA		NA		NA		NA		Y		NA		NA	Y	NA
**Sensitivity**: (c) Are analyses done on relevant subgroups?						N		P		N		P		N		N		N		N		P		N	P	N
**Results reporting**																										
**Cost sector**: Are the results presented transparently by cost category/sector?						Y		N		N		Y		Y		N		Y		Y		Y		Y	Y	N
**Generalizability**: Do the authors discuss the generalisability of study results (e.g. comparing the results to other patient/client groups/ or in other settings)?						Y		P		P		N		N		N		Y		N		P		N	P	N
**Limitations**: Do the authors discuss important limitations?						P		Y		P		Y		P		Y		Y		Y		Y		Y	P	Y
**Ethical and distribution issues**: Do the authors discuss ethical issues?						P		Y		P		Y		P		Y		Y		Y		P		Y	P	P
**Ethical and distribution issues**: Do the authors discuss distributional issues?						N		N		Y		N		N		N		Y		Y		P		N	P	P
**Conflict of interest**: Do the authors report any potential conflict of interest?						N		Y		Y		N		Y		N		Y		Y		Y		Y	Y	Y

Y = yes, N = no, P = partially, NA = not applicable, UC = unclear.

*This checklist was adapted from Schnitzler L, Roberts TE, Jackson LJ, Paulus ATG and Evers SMAA. A consensus-based checklist for the critical appraisal of cost-of-illness (COI) studies. *Int J Technol Assess Health Care*. 2023;39(1):e34. doi:10.1017/S026646232300019

**Table 5:** CHEERS 2022 reporting criteria of the included studies

Items	Rezende et al. [17]	Zheng et al. [18]	Zhao et al. [19]	O’Neill et al. [20]	Pakseresht et al. [21]	Shankar et al. [22]	Afkar et al. [23]	Afkar et al. [24]	Jalali et al. [25]	Mahmood et al. [26]	Lan et al. [27]	Umo et al. [28]
***Item 1.*** *Title: Identify the study as an economic evaluation and specify the interventions compared.*	Y	P	P	P	P	P	P	P	Y	P	P	Y
***Item 2***. *Abstract: Provide a structured summary that highlights context*,* key methods*,* results and alternative analyses.*	Y	Y	Y	Y	Y	Y	Y	Y	Y	Y	Y	Y
***Item 3.****Background and objectives: Give the context for the study*,* the study question and its practical relevance for decision-making in policy or practice.*	Y	Y	Y	Y	Y	Y	Y	Y	Y	Y	Y	Y
***Item 4.*** *Health economic analysis plan: Indicate whether a health economic analysis plan was developed and where available.*	Y	Y	Y	Y	Y	Y	Y	Y	Y	Y	Y	Y
***Item 5.****Study population: Describe characteristics of the study population (such as age range*,* demographics*,* socioeconomic*,* or clinical characteristics). Explanation.*	Y	Y	Y	Y	Y	Y	Y	Y	Y	Y	Y	Y
***Item 6.*** *Setting and location: Provide relevant contextual information that may influence findings.*	Y	Y	Y	Y	Y	Y	Y	Y	Y	Y	Y	Y
***Item 8.*** *Study perspectives: State the perspectives adopted by the study and why they were chosen.*	Y	Y	Y	Y	Y	Y	Y	Y	Y	Y	Y	Y
***Item 9.*** *Time horizon: State the time horizon for the study and why appropriate.*	P	P	N	P	Y	N	P	Y	P	N	Y	P
***Item 10.*** *Discount rate: Report the discount rate and reason chosen.*	N	N	N	N	N	N	Y	N	N	N	Y	N
***Item 14.*** *Measurement and valuation of resources and costs: Describe how costs were valued.*	Y	Y	P	Y	P	P	Y	Y	P	P	Y	P
***Item 15.****Currency*,* price date*,* and conversion: Report the dates of the estimated resource quantities and unit costs*,* plus the currency and year of conversion.*	Y	P	P	Y	P	P	P	Y	Y	P	Y	Y
***Item 18.*** *Characterising heterogeneity: Describe any methods for estimating how the study results vary for subgroups.*	N	N	N	N	N	N	N	N	Y	N	N	N
***Item 20.*** *Characterising uncertainty: Describe methods to characterise any sources of uncertainty in the analysis.*	N	N	N	N	N	N	N	Y	N	N	Y	N
***Item 21.****Approach to engagement with patients and others affected by the study: Describe any approaches to engage patients or service recipients*,* the general public*,* communities*,* or stakeholders (e.g.*,* clinicians or payers) in the study’s design.*	N	N	N	Y	Y	N	N	N	N	Y	N	N
***Item 22.****Study parameters: Report all analytic inputs (e.g.*,* values*,* ranges*,* references)*,* including uncertainty or distributional assumptions).*	N	N	N	N	N	N	N	P	P	P	Y	P
***Item 23.*** *Summary of main results: Report the mean values for the main categories of costs and outcomes of interest and summarise them in the most appropriate overall measure.*	N	N	N	Y	Y	P	Y	Y	Y	P	Y	P
***Item 24.****Effect of uncertainty: Describe how uncertainty about analytic judgments*,* inputs*,* or projections affects findings. Report the effect of the choice of discount rate and time horizon*,* if applicable.*	N	P	N	P	P	P	Y	Y	N	N	Y	N
***Item 25.****Effect of engagement with patients and others affected by the study: Report on any difference patient/ service recipient*,* general public*,* community*,* or stakeholder involvement made to the approach or findings of the study.*	P	P	P	P	P	P	P	P	N	P	P	N
***Item 26.****Study findings*,* limitations*,* generalisability*,* and current knowledge: Report key findings*,* limitations*,* ethical or equity considerations not captured*,* and how these could affect patients*,* policy or practice.*	P	Y	P	Y	P	Y	Y	Y	Y	Y	P	P
***Item 27.****Source of funding: Describe how the study was funded and any role of the funder in the identification*,* design*,* conduct and reporting of the analysis.*	Y	Y	Y	Y	N	N	N	Y	Y	N	Y	N
***Item 28.*** *Conflicts of interest: Report the author’s conflicts of interest according to journal or International Committee of Medical Journal Editors (ICMJE) requirements.*	N	Y	Y	N	Y	N	Y	Y	Y	Y	Y	Y

^CHEERS, the Consolidated Health Economic Evaluation Reporting Standards^

### Critical evaluation and quality reporting of included studies

The critical evaluation reports of the selected studies are reported in Table 4. Table 5 further provides a list of the CHEERS 2022 criteria in these studies.

The critical appraisal of the included studies using the consensus-based guidelines by Schnitzler et al. [10] in Table 4 found studies with varied quality and reporting styles. The majority of the studies lacked clear mentions or justifications for the time horizon and discount rates employed. The same was seen with sensitivity analysis and a few other parameters. Most of the studies stated the measurement of resources used, with only half of the studies providing a valuation of each unit price of the cost components used.

A few observations from the CHEERS 2022 criteria against the included studies are reported in Table 5. Item 1 of CHEERS 2022 criteria recommended for studies to mention ‘economic evaluation’ or related terms to ease the search for health economic studies. Zheng et al. [18] used the term ‘medical expenditure’ in the title, Pakseresht et al. [21]. used the term ‘expenditure’, Afkar et al. [24]. utilised the term ‘hospitalisation costs’, while Zhao et al. [19] included ‘cost based’ in the title. Rezende et al. [17]. were the closest to meeting this requirement by including the term ‘economic burden’ in their title.

Item 9 of CHEERS 2022 criteria described the time horizon of economic evaluation studies referring to the length of time over which costs were evaluated and reported. Since the time horizons for Rezende et al. [17], Zheng et al. [18], O’Neill et al. [20], Shankar et al. [22] and Afkar et al. [24] were not specified, it can be assumed that these studies gathered cost information within the treatment cycles. The studies did employ discount rates in their cost analysis, although the treatment cycles could range over more than a year.

Item 14 required studies to describe the measurement and valuation of resources and costs. However, Parseresht et al. [21] and Shankar et al. [22] reported costs based on categories of ranges without means or median. The other studies included means in their cost analysis. Several studies did not mention the price dates of the resources and currency conversion years of the study, which left the only assumption that the authors adopted the costs for the year(s) in which the study data were collected.

From the CHEERS 2022 assessment of the included studies in this review, it was discovered that most of the studies did not cover studies uncertainty, which is usually measured as sensitivity analysis in COI studies [16]. Similarly, only a few studies described the engagement with patients or other stakeholders and the effects of such engagement. It was also noted that not all studies included a description of study limitations, source of funding and conflict of interest of the authors, which are items to be reported according to CHEERS 2022.

#### Interventions to reduce cost of illness

None of the selected papers detailed a discussion on the intervention to reduce the costs of breast cancer.

#### Funding information of the included studies

All the studies in this systematic review reported the source of funding, except Pakseresht et al. [21], Shankar et al. [22], Afkar et al. [24] and Mahmood et al. [26]. None of the studies described the funder’s involvement in the identification, development, execution and reporting of the analysis. Reporting financial support in a study helps to clarify potential bias in the study.

## Discussion

Twelve COI studies from middle-income countries were identified with no study coming from low-income countries (LICs). Direct medical costs with treatment costs, diagnosis costs, lab investigations, outpatient or follow-up fees, and hospitalisation costs were reported but varied among the studies. Meanwhile, medications and hospitalisation contributed greatly to the total direct medical costs in these studies, which is not surprising.

Al-Ziftawi et al. [29] noted that breast cancer medications increased the economic burden of patients while these drugs may not be cost-effective in developing countries. An example was provided wherein trastuzumab was shown to be cost-effective in studies conducted in China and Taiwan but not in research conducted in Iran. Discussion was made about the possible genetic make-up of Asian women in the response to drug therapy. Kumar et al. [30] reported the economic burden of targeted drug therapy in breast cancer and the choices of drugs based on hospital formulary. Both researches highlighted the worries that rising cancer medication costs might undermine the ability to access cancer care.

Direct non-medical costs of transportation, lodging and food were reported in a few studies, while productivity loss was the only indirect cost reported in two studies. It was noted by Afkar et al. [23] that more advanced-stage patients were referred to public hospitals, thus increasing the mortality rates and resulting in much higher productivity loss compared to patients attending private hospitals.

Three studies did not report separate cost components. It is not surprising that many of the studies used an incidence-based study design with a retrospective and healthcare payers’ perspective. It is important to mention that only a small percentage of the included studies disclosed sensitivity analysis, discount rates and time horizon, which raises concerns about the reporting quality.

World Bank Report on Disease Control Priorities in Developing Countries (DCP) highlighted the differences in the availability of cancer treatment in LMICs and HICs [31]. Middle-income countries were further categorised as lower-middle and upper-middle-income countries. According to the DCP, LMICs have up to two times fewer surgical facilities and lower access to cancer treatment compared to UICs. Research in COI studies and breast cancer early detection are much needed in LMICs. More research funds to LMICs for COI and health economics studies enhance further understanding of breast cancer treatment and early detection strategies in these countries.

Decision-makers in the public and private sectors increasingly rely on COI studies to provide information on the costs incurred on illnesses affecting patients and a country’s healthcare resources [11]. COI studies provide important data for further cost-effectiveness studies into designing proper breast cancer treatment and screening programmes [32–35]. Methodological differences across COI studies may render them difficult to interpret and may be constrained by a lack of report transparency. These emphasise how critical it is for COI studies to report costs and cost aggregation. In addition, sensitivity analysis should be deployed in COI studies to accurately reflect the uncertainties affecting cost estimates [11].

The results indicated that the majority of COI studies in this review did not specify the cancer stages and the costs segregation by cancer stages. Numerous studies did not indicate the costs of treatment modalities, which left out important information as cost data by breast cancer stages could provide further information to decision-makers on the assessment of health services and allocating health resources. Dvortsin et al. [36] reported that treatment with cetuximab, bosutinib and trastuzumab was many folds more expensive and not cost-effective in late-stage breast cancer when compared to early stages. Other researchers discussed the importance of costs by cancer stages as it was reported that budget constraints in LMICs may restrict the accessibility of patients to treatment options of lower costs and ‘conservative’ compared to the higher priced ‘innovative’ treatment [11, 31]. The considerations of costs by cancer stages are to be factored-in when designing or reviewing COI studies. As considered in this study, cost types and disaggregation should be reported in all COI and cost analysis studies to provide useful and detailed information to the stakeholders. Cost disaggregation and components could be reported alongside each cost type to provide meaningful data.

The societal perspective on COI offers a more comprehensive view of the overall costs of illness to society. [9] It is more difficult and time-consuming to collect data from the societal perspective compared to the more common payer perspective. [9]The perspective adopted by a COI study should be aligned with the research questions and objectives of the study. The rationale for adopting certain perspectives in a study should be clarified and justified early in the study design.

The consensus-based guidelines by Schnitzler et al. [10] (Table 4) reported the lack of quality and details in the majority of the included studies, which was further seen across parameters like measurement and valuation of resources, time horizon, discount rates and sensitivity analysis. [16]A study’s time horizon and discount rates, together with sensitivity analysis, are important consideration factors when reviewing a COI study for decision-making [9]. It is challenging to assess the COI among the included studies when the time horizons were not precisely specified and varied among the studies in this systematic review. Healthcare resources in the future are valued at a lower rate than the present time, as costs have a time factor [9]. Furthermore, not employing discount rates in studies for more than a year reduces the reliability of the cost data analysis. Therefore, it is advisable to incorporate sensitivity analysis to examine the high and low estimates of healthcare costs in COI studies based on the usual range of discount rates from 3 to 5% [9]. Less than half of the included studies in this review conducted a sensitivity analysis with discount rates. Future studies could be planned with these included.

In terms of reporting the quality of the health economic studies as detailed in CHEERS 2022 [16], item 1 in the statements emphasised the importance of including ‘economic evaluations’ in the title to ease the process of searching and identifying such articles. Where relevant, the interventions being investigated in the study are to be mentioned. However, most studies in this review did not use identifiable terms such as ‘economic burden’, ‘cost of illness’ or related terms in the title. ‘Cost analysis’, ‘economic costs’, ‘out-of-pocket expenses’, ‘healthcare costs’ and ‘cost based’ were some of the other terms used by the LMIC studies in the title. A few studies mentioned health economic related terms not in the title, but in the abstract or content. The inconsistent use of terms in the title of health economics studies also occurred in studies from higher-income countries [35, 37–39]. Thus, authors are to keep in mind choosing an effective and inclusive title for COI studies to enhance the searchability of the study by others.

Item 14 of the criteria required resources and costs to be measured and valued, including cost types and cost disaggregation [11]. COI studies that do not describe the resource measurement and cost valuation render the results less useful in cost analysis, making it more difficult to compare studies. To facilitate a more impartial comparison, the implementation of a standardised Cost of Illness (COI) guideline, including suggested cost components, would be beneficial. If determining overall costs is not feasible, providing guidelines for each cost component would still be of considerable assistance.

Sensitivity analysis is a mathematical model to examine the effects of simulated dependent and independent factors on COI [7]. These analyses could have provided information about potential scenarios for reducing COI. Future COI studies should include sensitivity analysis in cost analysis.

This review has several limitations. Among them are the challenges in locating COI research due to the usage of a wide variety of cost or health economic terms. It was challenging to compare COI studies directly when costs were reported without a clear indication of the base year of the cost data, along with varied cost types reported. Further noting that many cost-effectiveness analysis (CEA) or cost-utility studies (CUA) used previously published data in their COI costing and did not have detailed descriptions of the local COI data; the author team has decided to exclude these studies in looking for full original COI data in this review.

Due to time and resource limitations, this systematic review excluded non-English language papers, which could have caused indexing bias. It is recommended that future reviews should plan the needed resources to review non-English papers in the study design stage. On another note, the inclusion of only peer-reviewed articles in this systematic review ensured certain standards of science in the selected articles, but may have caused possible individual reviewer bias and certain level of inconsistency. However, it is believed that the selection from four different databases in this study helped to diversify the search for articles. Future reviews could enhance the search for further grey literature and unpublished data to ensure the representativeness of included articles.

## Conclusion

Breast cancer mortality and incidence rates are rising in LMICs. COI studies provide insights into the healthcare usage among the countries and provide useful information to stakeholders in designing proper breast cancer treatment and screening programmes. This study found that direct medical costs are the highest costs compared to other cost types. Hospitalisation and medication costs further added to the burden of direct medical costs. Direct non-medical costs and productivity losses place a further financial burden on breast cancer patients. COI studies, which include cost types and cost disaggregation, provide more useful data for decision-making. These components should be in the research design of future COI studies. Consensus Guidelines for the Critical Appraisal of COI studies and CHEERS 2022 statements recommended reporting the uncertainty of health economic studies through sensitivity analysis. Studies which did not report sensitivity analysis and discount rates should be read carefully in context. Future COI research should analyse the association between cost components and other variables in accounting for data disparities to the factors.

### Electronic supplementary material

Below is the link to the electronic supplementary material.

Supplementary Material 1

## Data Availability

Not applicable.
