# Supplementary material for: Cost of illness of breast cancer in low- and middle-income countries: a systematic review
Source: Health Econ Rev. 2024 Jul 22;14:56. doi: 10.1186/s13561-024-00536-0 (PMC11264967; doi:10.1186/s13561-024-00536-0)
Supplement: Supplementary file 1 — Supplementary Material 1 [file 13561_2024_536_MOESM1_ESM.docx]

**Search Report**

**TITLE: Cost of Illness of Breast Cancer in Low- and Middle-Income Countries: A Systematic Review**

**Time frame: literatures until 25 Dec 2023**

| **Source** | **Date of Search** | **Records retrieved** |
| --- | --- | --- |
| 1. COCHRANE Library | 15/5/2022, 12/12/2022 & 25/12/2023 | 26 |
|  |  |  |
| 2. Proquest Dissertation & Thesis | 12/12/2022 & 25/12/23 | 20 |
|  |  |  |
| 3. Scopus Database | 15/5/2022, 12/12/2022 & 25/12/2022 | 881 |
|  |  |  |
| 4. Pubmed | 15/5/2022, 12/12/2022 & 25/12/2023 | 424 |
|  |  |  |
| **Total before de-duplication** | | 1351 |
| **Total after de-duplication** | | 1017 |

| Title of Systematic Review | **Cost of Illness of Breast Cancer in Low- and Middle-Income Countries: A Systematic Review** | |
| --- | --- | --- |
| Identification | Records identified through database searching | 1351 |
|  | Additional records identified through other sources | 2 |
| Screening | Records after duplicates removed | 1017 |
|  | Records screened | 1017 |
|  | Records excluded | 971 |
| Eligibility | Full-text articles assessed for eligibility | 46 |
|  | Full-text articles excluded, with reasons | 34 |
| Included | Studies included in qualitative synthesis | 12 |
|  | Studies included in quantitative synthesis (meta-analysis) | 0 |
